# A Three-Dimensional Imaging Method for the Quantification and Localization of Dynamic Cell Tracking Posttransplantation

**DOI:** 10.3389/fcell.2021.698795

**Published:** 2021-09-07

**Authors:** Fengfeng Lu, Xin Pan, Wencheng Zhang, Xin Su, Yuying Gu, Hua Qiu, Shengwei Shen, Changcheng Liu, Wei Liu, Xicheng Wang, Zhenzhen Zhan, Zhongmin Liu, Zhiying He

**Affiliations:** ^1^Institute for Regenerative Medicine, Shanghai East Hospital, School of Life Sciences and Technology, Tongji University, Shanghai, China; ^2^Shanghai Engineering Research Center of Stem Cells Translational Medicine, Shanghai, China; ^3^Shanghai Institute of Stem Cell Research and Clinical Translation, Shanghai, China; ^4^Department of Cardiology, Shanghai East Hospital, Tongji University, Shanghai, China; ^5^The First Affiliated Hospital of Nanchang University, Nanchang, China; ^6^Department of Hepatobiliary and Pancreatic Surgery, Shanghai East Hospital, School of Medicine, Tongji University, Shanghai, China; ^7^Institute of Heart Failure, Shanghai East Hospital, Tongji University, Shanghai, China

**Keywords:** cell transplantation, *in vivo* tracking, bioluminescence imaging (BLI), computer tomography (CT), cell distribution

## Abstract

Cell transplantation has been proposed as a promising therapeutic strategy for curing the diseases requiring tissue repairing and functional restoration. A preclinical method to systematically evaluate the fates of donor cells in recipients, spatially and temporally, is demanded for judging therapeutic potentials for the particularly designed cell transplantation. Yet, the dynamic cell tracking methodology for tracing transplanted cells *in vivo* is still at its early phase. Here, we created a practical protocol for dynamically tracking cell *via* a three-dimensional (3D) technique which enabled us to localize, quantify, and overall evaluate the transplanted hepatocytes within a liver failure mouse model. First, the capacity of 3D bioluminescence imaging for quantifying transplanted hepatocytes was defined. Images obtained from the 3D bioluminescence imaging module were then combined with the CT scanner to reconstruct structure images of host mice. With those reconstructed images, precise locations of transplanted hepatocytes in the liver of the recipient were dynamically monitored. Immunohistochemistry staining of transplanted cells, and the serology assay of liver panel of the host mice were applied to verify the successful engraftment of donor cells in the host livers. Our protocol was practical for evaluating the engraftment efficiency of donor cells at their preclinical phases, which is also applicable as a referable standard for studying the fates of other transplanted cells, such as stem cell-derived cell types, during preclinical studies with cell transplantation therapy.

## Introduction

Today, cell transplantation therapy plays important roles in clinical therapies for many types of diseases. Since 1967, the well-known success of bone marrow transplantation had started a new era of cell transplantation for the therapies of blood diseases (Thomas, 1987, 2005; Cossu et al., 2018). Later, hepatocyte transplantation was also successfully used in the clinical trials for some liver diseases (Ott and Castell, 2019). The current studies in the fields of stem cells and regenerative medicine are hoped to provide the adequate sources of donor cells and bring the cell transplantation therapy into even newer era, which have widely opened the scope to forecast its potential applications into therapies of many injuries or diseases for tissue repairing or replacements. Especially, stem cell-derived functional cells for various tissues are increasingly considered sources of donor cells for cell transplantation therapy in the future.

To evaluate the therapeutic effects of cell transplantation, the dynamic migration and movements of donor cells in the hosts are required to be comprehensively analyzed during preclinical studies. Therefore, a practical method to track the state and fate of donor cells posttransplantation spatially and temporally is highly demanded in many current and future studies on the preclinical applications of cell transplantation-based therapies (Nguyen et al., 2014).

The dynamic study with comprehensive analyses on transplanted cells through using the new technology of *in vivo* tracking has become a powerful strategy to judge the fate of transplanted cells (Feigenbaum et al., 2009). Such new technology is under a non-invasive condition, which does not require the multiple rounds of sacrificing animals and harvesting samples. Especially, non-invasive tracking has many common advantages to perform the dynamic cell tracking in a real-time process, which were able to monitor the fate of transplanted cells *in vivo* during various dynamic processes, including the stages of cell homing and engraftments, as well as target tissue repairing or replacements for functional recoveries of many tissues (Kircher et al., 2011).

Bioluminescence imaging (BLI) technique system carries many potential advantages for dynamic tracking on cells *in vivo*. Most used bioluminescence is firefly luciferase, with Mg^2+^, ATP, and O_2_, which catalyze its substrate luciferin to generate bioluminescence signals. Luciferase has been used as a reporter gene product to recognize those luciferase-expressing cells during their biological processes (Dubey, 2012). With a set of optical inspection instruments, dynamic activities of the transplanted luciferase-expressing cells in the body of recipients can be directly monitored, based on the luciferase-induced bioluminescence signal (Herter-Sprie et al., 2014).

Normally, BLI could only be used at the mode of two dimensions (2D), which was successfully applied in the studies on observations of tumor growth *in vivo* (Yao et al., 2018). However, BLI of 2D mode (2D BLI) could not reach to the enough depths of tissues to track signals of detected cells that located deeply in the body of recipients (Kocher and Piwnica-Worms, 2013). Recently, BLI of 3D mode (3D BLI) has been developed with significant improvements, which enable scientists to capture both the information more accurately about localization and quantification of donor cells during the dynamic cell tracking after cell transplantation.

Based on the latest generation of instrument for IVIS^®^ Spectrum computed tomography (CT) scanner that combines optical imaging and CT in one platform (Aalipour et al., 2019), we investigated how to establish a practical protocol to perform 3D cell tracking of transplanted hepatocytes in mice. This protocol was also expected to be a useful and referable standard for the 3D tracking of other cell types during their dynamic process of cell transplantation.

In our study, the fumarylacetoacetate hydrolase-deficient (*Fah^–/–^*) mouse model was used as the preferable system for hepatocyte transplantation and liver repopulation. *Fah^–/–^* mice can undergo induced liver failure and animal death once lacking enough 2-(2-nitro-4-trifluoromethylbenzoyl)-1,3-cyclohexanedione (NTBC) water. However, they can also be successfully rescued from death by restoring their liver function through the liver repopulation after transplantation of hepatocytes from wild-type animals. In our experiment, the dynamic process, including the stages of cell engraftment, migration, proliferation, and liver regeneration in final, was continuously tracked in a real-time manner after transplanting luciferase-expressing hepatocytes into the body of *Fah^–/–^* mouse.

## Method

### Animals

All mice received humane care according to the guidelines of Tongji University Animal Care and Use Committees. Luciferase transgenic mice were purchased from Shanghai Model Organisms Center, Inc., maintained under specific pathogen-free conditions and used at 8–10 weeks of age.

### Mice Model With Liver Failure

As described in previous studies (He et al., 2010; Wang et al., 2018), *Fah^–/–^* mice undergo liver failure and death. To survive, the mice need to be maintained with continuous supplement of 2-(2-nitro-4-trifluoromethylbenzoyl)-1,3-cyclohexanedione (NTBC) in their drinking water (7.5 mg/L). To induce liver injury, NTBC was totally withdrawn from the drinking water of *Fah^–/–^* mice.

### Hepatocyte Isolation and Transplantation

Donor luciferase-expressing hepatocytes were isolated from luciferase transgenic mice (Shanghai Model Organisms Center, Inc., Shanghai, China) with the perfusion protocol that is established in previous studies (He et al., 2012; Wang et al., 2014). Briefly, the liver was preperfused with 1 × Earle’s balanced salt solution (EBSS) (Gibco, Amarillo, TX, United States) with 5 mM 4-(2-hydroxyethyl)-1-piperazineethanesulfonic acid (HEPES) (Gibco) for 10 min at 37°C, and then perfused with 1 × EBSS with 5 mM HEPES with collagenase D (Sigma-Aldrich, St. Louis, MO, United States) for 10 min at 37°C. The dissociated cells were filtered through a 70-μm Nylon cell strainer (BD Biosciences, San Jose, CA, United States), and then centrifuged twice for 2 min at 50 g to remove clumps. Furthermore, viability of isolated hepatocytes was above 80% evaluated by Trypan blue stain (Sigma-Aldrich). Harvested hepatocytes were resuspended in DMEM and injected into *Fah^–/–^* recipient animals through the spleen (1 × 10^6^ cells/recipient) as described (Wang et al., 2014).

### CT for Three-Dimensionally Localizing Luciferase-Expressing Hepatocytes

Computed tomography scanning was carried out with IVIS^®^ Spectrum CT *in vivo* Imaging System (PerkinElmer, Waltham, MA, United States). CT images were captured with 50 kVp X-rays at a tube current of 1 mA, with exposure time of 50 ms, and with an aluminum filter. CT images were reconstructed with Living Image 4.5 software, which not only provided a field of view (FOV) of 20 cm × 20 cm × 20 cm but also allowed an isotropic resolution of 0.15 mm for the anatomical location of transplanted cells with bioluminescence.

### Bioluminescence Imaging of Transplanted Hepatocytes

#### *In vitro* BLI of Luciferase-Expressing Hepatocytes in 2D System

The isolated luciferase-expressing hepatocytes were seeded in 96-well plates with different densities (1 × 10^3^, 1 × 10^4^, and 1 × 10^5^). After being attached in the CO_2_ incubator for 4 h under 37°C, the value of bioluminescence excited by cells was measured for depleting the background signals. D-Luciferin (250 μM) was then added to the attached hepatocytes in the 96-well plate. After reacting for 5 min, the plate with cells was placed in the IVIS^®^ Lumina III *In Vivo* Imaging System (PerkinElmer) for detecting the intensity of bioluminescence. The imaging data were analyzed by Living Image software to determine correlations between signal intensity (p/s/cm^2^/sr) and cell numbers.

#### *In vivo* BLI of Luciferase-Expressing Hepatocytes in Host Mice After Transplanting

Bioluminescence imaging of *Fah^–/–^* mice were captured at different intervals after transplantation (3 days, 1, 3, 5, 8, and 10 weeks). To conduct the BLI, host *Fah^–/–^* mice were intraperitoneally injected with D-luciferin (100 mM) right after anesthesia. After reaction for 5 min (for 2D BLI) or 8 min (for 3D BLI), the mice were placed in the light-tight chamber in the IVIS^®^ Spectrum CT *in vivo* Imaging System. Dorsal imaging was performed for all host mice at different time points. For 2D BLI, no emission filter was used during the imaging process. For 3D BLI, five bandpass open emission filters were centered at 560, 580, 600, 620, and 640 nm to collect the optical signal, respectively.

The imaging signals collected within 5 min were used to locate the transplanted luciferase-expressing hepatocytes under 3D BLI, and the density of proliferating cell groups or nodules could be distinguished by different colors intuitively. Those colors indicated diverse intensity level and corresponding color scale at the right side of every picture. Beforehand, the reconstructed 3D whole-body structure image of *Fah^–/–^* mouse recipient was captured by CT scanner. By combining the 3D BLI with CT-reconstructed whole-body structure images, the results of the real-time distributions of transplanted luciferase-expressing hepatocytes in the liver and other organs of each *Fah^–/–^* mouse recipient were collected for further analysis. FOV = 13.2 × 13.2 cm, f/stop = 1, binning = 8.

#### Analysis of BLI

Bioluminescence signal intensity was calculated by Living Image 4.5 Software (PerkinElmer). The photon radiance was measured as photons per second per square centimeter per steradian (p/s/cm^2^/sr) within the regions of interest (ROIs) (Kuo et al., 2007; Thompson et al., 2013). Bioluminescence unit was showed as total flux radiance which is a calibrated measurement based on photon emission.

### Real-Time Quantitative PCR

Real-time quantitative PCR (RT-qPCR) was performed with the TB Green Premix Ex Taq II (Takara, Kusatsu, Japan). The expression of glyceraldehyde 3-phosphate dehydrogenase (GAPDH) was used as control. PCRs were performed under the following conditions: 95°C for 15 s followed by 40 cycles at 95°C for 5 s, 60°C for 34 s, 95°C for 1 min, and 55°C for 1 min. The primers used for the assay include luciferase-Forward 5′-TTACCAGGGATTTCAGTCG-3′; luciferase-Reverse 5′-CCTT TAGGCAGACCAGTAGA-3′; GAPDH-Forward 5′-AGGTCGG TGTGAACGGATTTG-3′; GAPDH-Reverse 5′-GGGGTCGTT GATGGCAACA-3′.

### Immunohistochemistry Assay

The transplanted *Fah^–/–^* mice were sacrificed at experimental time points. The liver samples were harvested, and immunohistochemistry with FAH antibody was conducted to examine the percentage of liver repopulations. For immunohistochemistry staining, the fresh liver samples were fixed in 4% paraformaldehyde, embedded in paraffin, and cut into 2-μm-thick slices. Sections were baked, deparaffinized, rehydrated, permeabilized with 3% H_2_O_2_ solution for 15 min, and blocked by 1% BSA for 30 min. The sections were then incubated with rabbit antimouse FAH primary antibody (HepatoScience, 1:3,000) at 4°C overnight and with secondary antibodies conjugated with HRP (Sangon Biotech) at 37°C for 30 min. After staining with DAB (MXB) in proper intensity, the sections were counterstained with hematoxylin (Beyotime, Jiangsu, China), dehydrated rapidly, and mounted with neutral resin (SolarBio, Beijing, China). Finally, the samples were examined under optical microscope (Leica, Wetzlar, Germany).

Aperio ImageScope software of pathological section scanner (Leica SDPTOP HS6) was served for immunohistochemistry stain imaging and statistics of FAH^+^ area and liver area. We picked no less than three discontinuous sections in each liver to calculate FAH^+^ area and liver area. In the analysis, regions of interest (ROIs) were calculated by software automatically.

### Statistical Analysis

Data are presented as mean ± SD or mean ± SEM from at least three independent experiments unless otherwise indicated. One-way ANOVA with Tukey’s *post hoc* test was used for multiple groups and Student’s *t*-test for two groups. *p* < 0.05 was considered statistically significant (^∗^*p* < 0.05, ^∗∗^*p* < 0.01, ^∗∗∗^*p* < 0.001. *N* = 3).

## Results

### A Positive Linear Correlation Between Bioluminescence Intensity and Number of Luciferase-Expressing Hepatocytes Is Established Both *in vitro* and *in vivo*

Donor hepatocytes expressing luciferase were obtained from the luciferase transgenic mouse generated from our previous study (Wangensteen et al., 2008). Expression of luciferase enabled liver cells to be detected based on the bioluminescence released from biochemical induction of luciferase with luciferin (Supplementary Figure 1A). The viability of luciferase-expressing hepatocytes was evaluated based on vitality and healthy morphology compared with healthy wild-type hepatocytes. The correlation between the level intensity of induced bioluminescence releasing and the number of luciferase-expressing hepatocytes was determined (Supplementary Figure 1B). Results indicated that the total flux of bioluminescence intensity for 1 × 10^3^, 1 × 10^4^, and 1 × 10^5^ of hepatocytes were 6.222 × 10^5^, 1.255 × 10^7^, and 5.5265 × 10^7^ p/s/cm^2^/sr, respectively (Supplementary Figure 1A). The results revealed a positive linear correlation between bioluminescence intensity and number of luciferase-expressing hepatocytes existed (*R*^2^ = 0.9959) (Supplementary Figure 1B). In the calculated averages, total bioluminescent photons emitted by each luciferase-expressing hepatocyte were approximately 8.152 × 10^2^ p/s/cm^2^/sr.

Then, luciferase-expressing hepatocytes were transplanted into *Fah^–/–^* mice with acute liver failure *via* spleen injection (1 × 10^6^ hepatocytes/mouse). For monitoring the process of surviving, engrafting, and proliferating of transplanted luciferase-expressing hepatocytes, 2D BLI on *Fah^–/–^* mice was performed at different time points (3 days, 1, 3, 5, 8, and 10 weeks) after transplantation. Evident bioluminescence signal was observed in *Fah^–/–^* mice on the third day after transplantation, suggesting that the luciferase-expressing hepatocytes were successfully transplanted into *Fah^–/–^* mice recipients (Supplementary Figure 1C).

After a slightly decreasing in *Fah^–/–^* mice recipients at the first week posttransplantation, bioluminescence signals started to increase significantly at the third week posttransplantation. From the fifth-week to eighth-week posttransplantation, the bioluminescence signals in *Fah^–/–^* mice recipients increased continually (Krohn et al., 2009). Based on this linear correlation between the bioluminescence intensity and number of luciferase-expressing hepatocytes obtained from the above experiments, the number of luciferase-expressing hepatocytes in *Fah^–/–^* mice recipients were calculated by the measured bioluminescence values (Supplementary Figures 1D,E).

### Developing a 3D BLI and CT Combined Real-Time Localizing and Quantification Methodology for Tracking the Transplanted Hepatocytes in Livers of *Fah^–/–^* Mouse Recipients

Next, the combined methods of 3D BLI and CT were performed to make a dynamic tracking on the localization and quantification of transplanted luciferase-expressing hepatocytes. The same animal recipients used during above 2D BLI were further analyzed in the process of 3D BLI and the comparisons between 3D and 2D imaging were specially performed.

For 3D BLI, open emission filters were performed within a range of 560–640 nm (every 20 nm bandpass) to collect the optical signal for five times. Images that the signals collected within 5 min were used to locate and quantify the luciferase-expressing cells in the recipients in real time. Meanwhile, CT scanning was carried out to scan mouse stereo body to visually reconstruct their 3D internal structures. Thus, the distribution of transplanted hepatocyte *in vivo* could be presented vividly (Supplementary Movie 1).

**FIGURE 1:**
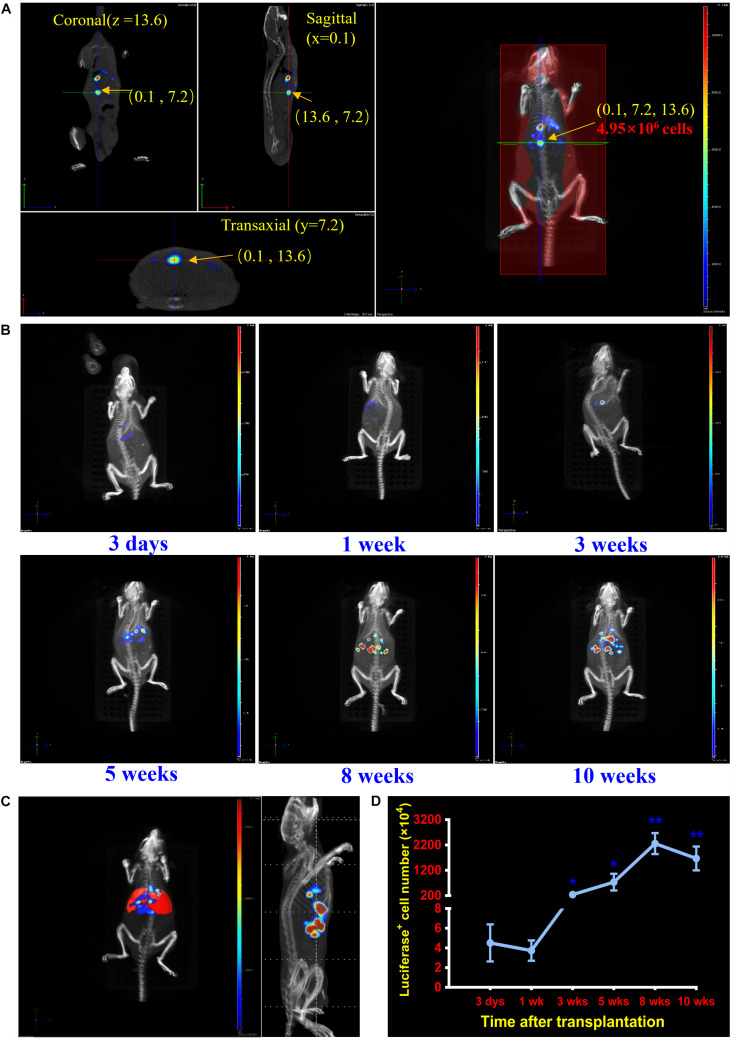
Localization and quantification analysis of transplanted cells in the liver of *Fah^–/–^* mice. **(A)** Establishment of the dynamic cell-tracking strategy for posttransplantation localizing and quantifying of grafted cells *in vivo*. A combination of 3D BLI and CT is established to locate and quantify the transplanted hepatocytes in livers of *Fah^–/–^* mouse recipients at any time point of detection. Representative image of accurate localization (0.1, 7.2, 13.6) cm and cell quantification (4.95 × 10^6^) of one nodule repopulated by transplanted hepatocytes (pointed by the arrow). **(B)** Dynamically localizing transplanted hepatocytes at different time points posttransplantation. Representative merged images of transplanted hepatocytes of *Fah^–/–^* mice at 3 days, 1, 3, 5, 8, and 10 weeks after hepatocyte transplantation. **(C)** Spectrum CT image of mouse liver was reconstructed by Living Image 4.5 software in *Fah^–/–^* mouse. **(D)** Quantification of transplanted hepatocytes in livers of *Fah^–/–^* mice at 3 days, 1, 3, 5, 8, and 10 weeks. Quantitative analyses of transplanted hepatocytes in liver of *Fah^–/–^* mice. Data are shown as mean ± SEM. *p < 0.05, **p < 0.01. *N* = 3. Student’s *t*-test.

During the process, the midpoint in every dimensional of recipient mouse was regarded as initial point. Every bioluminescent cell or cell-composed cluster *in vivo*, such as single luciferase-expressing repopulation nodules, could be positioned and analyzed on horizontal plane (*Z*), vertical plane (*Y*), and longitudinal plane (*X*), respectively (Supplementary Movie 2). Through these analyses, the transplanted luciferase-expressing hepatocytes could be precisely located (*x*, *y*, *z*) cm from the initial point at each detection time point. The total number could be quantified at the same detection time point according to the optical signal values of single hepatocyte/cluster with bioluminescence tomography (Figure 1A and Supplementary Movie 2). With the established protocol, luciferase-expressing hepatocytes were precisely localized in *Fah^–/–^* mice at six time points (3 days, 1, 3, 5, 8, and 10 weeks) after transplantation (Figure 1B and Supplementary Movie 1).

To further localize the numbers of cells engrafted in livers of *Fah^–/–^* mice at detection time points, a liver module was calculated and added to the images according to the parameters of the liver sizes and its distances to the abdominal surface (Figure 1C). With such module, engrafted numbers of luciferase-expressing hepatocytes were determined. As shown in the Figure 1D, on the third day of transplantation, less than 1 × 10^5^ hepatocytes could be successfully engrafted into the livers of *Fah^–/–^* mouse recipients. However, by 10 weeks after transplantation, the number of luciferase-expressing hepatocytes in *Fah^–/–^* mice expended to 1.84 × 10^7^. Of note, there are no consideration of degradation or maldistribution when using luciferase expression as a marker, and therefore a long-term and timelessness of cell tracking can be performed.

### Traditional Assessment of Posttransplantation Hepatocytes *via* 3D Cell Dynamic Tracking Process

Finally, to validate the results from 3D cell dynamic tracking process, the results from the traditional methods that are still routinely used in detection processes, involving conventional BLI imaging of isolated organs (liver, spleen, kidney), quantitative PCR test and the histochemical observations, were performed. In the liver of transplanted *Fah^–/–^* mice, BLI signal analysis showed the signal value was significantly improved at 10 weeks compared with that at 3 days. In contrast, the BLI value was significantly decreased in the spleen at 10 weeks compared with that at 3 days. There was no evident difference between the BLI values in the kidney of *Fah^–/–^* mice between 10 weeks and 3 days (Supplementary Figure 2A).

FAH protein, the donor cell-specific marker, was used to prove the existence of donor-derived hepatocytes in transplanted *Fah^–/–^* mice. To unravel the detailed number of regenerative hepatocytes, we assessed the regeneration efficiency *via* FAH^+^ area/liver area based on the immunohistochemistry staining of FAH antibody. The results showed that FAH^+^ hepatocytes was about 0.77% after 3 days posttransplantation, while it reached to approximately 80% after 10 weeks of repopulation (Supplementary Figures 2B,C). Consistently, the expression level of *luciferase* gene at 10 weeks was obviously higher than that at 3 days after cell transplantation (Supplementary Figure 2D).

To further validate regenerative status, we also examined liver functions of donor-derived hepatocytes grafted *Fah^–/–^* mice. Results showed that several parameters of liver function, including alanine aminotransferase (ALT), aspartate aminotransferase (AST), total bilirubin (T-BIL), and albumin (ALB), were recovered to the normal ranges at 10 weeks posttransplantation (Supplementary Figure 2E).

## Discussion

For the first time, a 3D BLI imaging system was established and was successfully used in combination with CT scanning to evaluate the dynamic process of *in vivo* cell tracking for the transplanted cells, which can precisely assess the localization and quantification of transplanted cells in the relative animal recipients.

Previously, several traditional methods had been developed for judging the transplanted donor cells during preclinical studies, which required enormous experimental animals while still only capable of conducting limited numbers of assays, and analyses for evaluating expected outcomes of cell transplantation. Moreover, these analytical processes could only rely on the multiple rounds of animal sacrificing and sample harvesting at limited time points after cell transplantations (Massoud and Gambhir, 2003). Therefore, a large number of animals were required for one study. In addition, these traditional methods also have chances of mistaken organ collections and misinterpretations due to inaccurate observations during necropsy and animal harvesting, partially because of the indirect or delayed interpretations for the real-time facts of those *in situ* and dynamic occurrences (Phair and Misteli, 2001). Therefore, the non-invasive tracking methods are crucial for achieving the goal of evaluating the cell fate posttransplantation.

Among several non-invasive tracking methods, the two representative ones used currently are magnetic particle imaging (MPI) and photoacoustic imaging (PAI). MPI is a method based on tomography for detecting the spatial distribution of superparamagnetic iron oxide nanoparticles (SPIONs) (Haegele et al., 2012; Wei et al., 2018). When labeled with Resovit, a particular type of SPIONs, a minimum of 200 mesenchymal stem cells can be successfully detected after transplantation during a tracking period up to 3 months (Bulte et al., 2015). However, both traceability and accuracy of SPIONs for MPI could be susceptibly influenced from gas, calcification, and blood flow in tissues (Bourquin et al., 2018; Zhou et al., 2018).

On the other hand, the method of PAI combines high optical contrast with high ultrasound resolution in a single imaging modality (Knox and Chan, 2018). During PAI analyses, the transplanted cells are labeled by the nanogold particles with photoacoustic effects. After transplantation, the nanogold particle-labeled cells could be dynamically tracked in real time by photoacoustic signal for 4 weeks (Jokerst et al., 2012; Zheng et al., 2015; Zhang et al., 2017). In similar with MPI, PAI also has some limitations. For instance, the nanomaterials used in PAI can be mainly distributed in the lysosomes after entering cells through endocytosis. Therefore, it may become difficult to ensure the equal distributions in the offspring cells derived from original donor cells that carry nanomaterials with such limitations (Knox and Chan, 2018).

Bioluminescence imaging is another currently used technology to detect the signal of luminescence light emitted from enzyme-catalyzed reactions. In our previous study, the 2D BLI was performed to measure numbers of repopulating hepatocytes during liver repopulation (Wangensteen et al., 2008). However, these results from 2D BLI could only provide the information on survival and proliferation tendency for the transplanted luciferase-expressing hepatocytes. The key information on localization and quantification of the transplanted luciferase-expressing hepatocytes in *Fah^–/–^* mice recipients could not be obtained under 2D BLI. Notably, the combined methods of both 3D BLI and CT developed in this study enabled us to make a dynamic tracking on the localization and quantification of transplanted luciferase-expressing hepatocytes. Here, 3D BLI was tested for its first use to dynamically monitor the transplanted luciferase-expressing hepatocytes in *Fah^–/–^* mice recipients in real time. In addition, our results indicated that the combined technologies with both 3D BLI and CT made it possible to monitor the spatial and temporal distribution of transplanted cells in recipient and to determine the exact number of cells in different distributive regions during cell transplantation assay. Furthermore, with our model system, a new analysis program for localization and quantitation was established for our following studies, to finalize a standard protocol for transplanting any luciferase-expressing cells when 3D BLI and CT were performed simultaneously.

Although our method has achieved the *in vivo* localization and quantification of transplanted hepatocytes, there are still some issues to be further addressed. Firstly, the detection sensitivity of the current system still needs to be improved. With our 3D dynamic cell tracking system, clusters of ∼50 cells can be detected, but it is not sufficient to localize and quantitatively detect a single cell. The establishment of AkaLuc mutagenesis from luciferase may help us to achieve this goal (Iwano et al., 2018). AkaLuc can pair with its optical substrate AkaLumine hydrochloride, which produces a roughly fourfold improvement in the expression level than luciferase. This ultra-enhanced signal enables scientists to non-invasively visualize single cells deep inside freely moving animals (Iwano et al., 2018). Secondly, the resolution of CT also needs to be improved. We are currently improving our resolution by establishing a Bruker micro-CT-based system, which enable us to convert images into a complete microscopic visualization solution with the specialized software for further analysis. Finally, the *in vivo* depth of transplanted cells currently established for detection is limited. Our method is commonly used for posttransplant internal localization in small animals such as mice and rats; the application of the system on large animal or in human will be tested in the future study.

Taken together, the combination of 3D BLI and CT scanning could be used to analyze transplanted hepatocytes on several parameters in real time, including cell distribution, quantification, and location. Significantly, a protocol for such study process could be applied to judge whether the donor hepatocytes were qualified candidates for the potential clinical cell transplantation to liver failure patients, on considering both safety, and therapeutic efficiency. In addition, our established protocol is also expected to become a valuable and referable standard for visually analyzing other cell candidates, which were used for transplantation therapies in any kinds of animal models during their preclinical studies (He et al., 2010).

## Data Availability Statement

The original contributions presented in the study are included in the article/Supplementary Material, further inquiries can be directed to the corresponding author/s.

## Ethics Statement

The animal study was reviewed and approved by Tongji University Animal Care and Use Committees.

## Author Contributions

FL and XP conducted most of the experiments and imaging analysis. XS, YG, HQ, SS, and CL conducted the *Fah^–/–^* mice management and hepatocytes grafting with FL and XP. WL and ZZ conducted the *in vitro* assays which were provided as controls of this study. ZH designed the study. ZH and ZL supported and supervised the study. ZH, FL, WZ, XP, and XW wrote the manuscript with help from the other authors. All authors contributed to the article and approved the submitted version.

## Conflict of Interest

The authors declare that the research was conducted in the absence of any commercial or financial relationships that could be construed as a potential conflict of interest.

## Publisher’s Note

All claims expressed in this article are solely those of the authors and do not necessarily represent those of their affiliated organizations, or those of the publisher, the editors and the reviewers. Any product that may be evaluated in this article, or claim that may be made by its manufacturer, is not guaranteed or endorsed by the publisher.

## Supplementary Material

Supplementary Figure 1 Analysis of bioluminescence intensity of luciferase hepatocytes *in vitro* and *in vivo* after transplanting into *Fah^–/–^* mice. **(A)** BLI image of luciferase hepatocytes *in vitro*. **(B)** The linear correlation assay between the number of luciferase hepatocytes and BLI signal intensity, respectively. **(C)** Representative pictures of 2D BLI imaging of *Fah^–/–^* mice (3 days, 1, 3, 5, 8, and 10 weeks). **(D)** Quantified analysis of 2D BLI signal intensity of *Fah^–/–^* mice *in vivo*. **(E)** Quantified analysis of transplanted hepatocytes in 2D of *Fah^–/–^* mice *in vivo*. Data are shown as mean ± SEM. ^∗^*p* < 0.05, ^∗∗^*p* < 0.01, ^∗∗∗^*p* < 0.001. *N* = 3. Student’s *t*-test.

Supplementary Figure 2 Assessment of posttransplantation hepatocytes in *Fah^–/–^* mice. **(A)** Bioluminescence intensity of isolated livers, spleen, kidney of *Fah^–/–^* mice, respectively. **(B)** Immunohistochemistry staining of histological sections of the livers from *Fah^–/–^* mice with luciferase-hepatocyte transplantation. *N* = 3. Scale bars, 200 μm (up) and 50 μm (down). **(C)** Quantification analysis for liver regeneration of *Fah^–/–^* mice at 3 days and 10 weeks after transplantation, respectively. **(D)** The relative expression level of luciferase gene in the liver of *Fah^–/–^* mice. **(E)** Serum levels of ALT **(a)**, AST **(b)**, ALB **(c)**, and total bilirubin **(d)** in *Fah^–/–^*, *Fah^–/–^* + luciferase hepatocytes *(Fah^–/–^* + Luc-Heps). Data are presented as mean ± SD. ^∗^*p* < 0.05, ^∗∗^*p* < 0.01, ^∗∗∗^*p* < 0.001. *N* = 3. Student’s *t*-test.

Supplementary Movie 1 3D-BLI signal intensity of *Fah^–/–^* mice after transplantation. Real-time 3D BLI imaging and CT scanning of transplanted hepatocytes in *Fah^–/–^* mice at 3 days, 1, 3, 5, 8, and 10 weeks after transplantation, respectively.

Supplementary Movie 2 Localization analysis of transplanted cells. 3D localization analysis of transplanted cells from horizontal (*Z*), vertical (*Y*), and longitudinal plane (*X*) *in vivo*.
