# Supplementary figures and images for: A Three-Dimensional Imaging Method for the Quantification and Localization of Dynamic Cell Tracking Posttransplantation
Source: Front Cell Dev Biol. 2021 Sep 7;9:698795. doi: 10.3389/fcell.2021.698795 (PMC8452970; doi:10.3389/fcell.2021.698795)

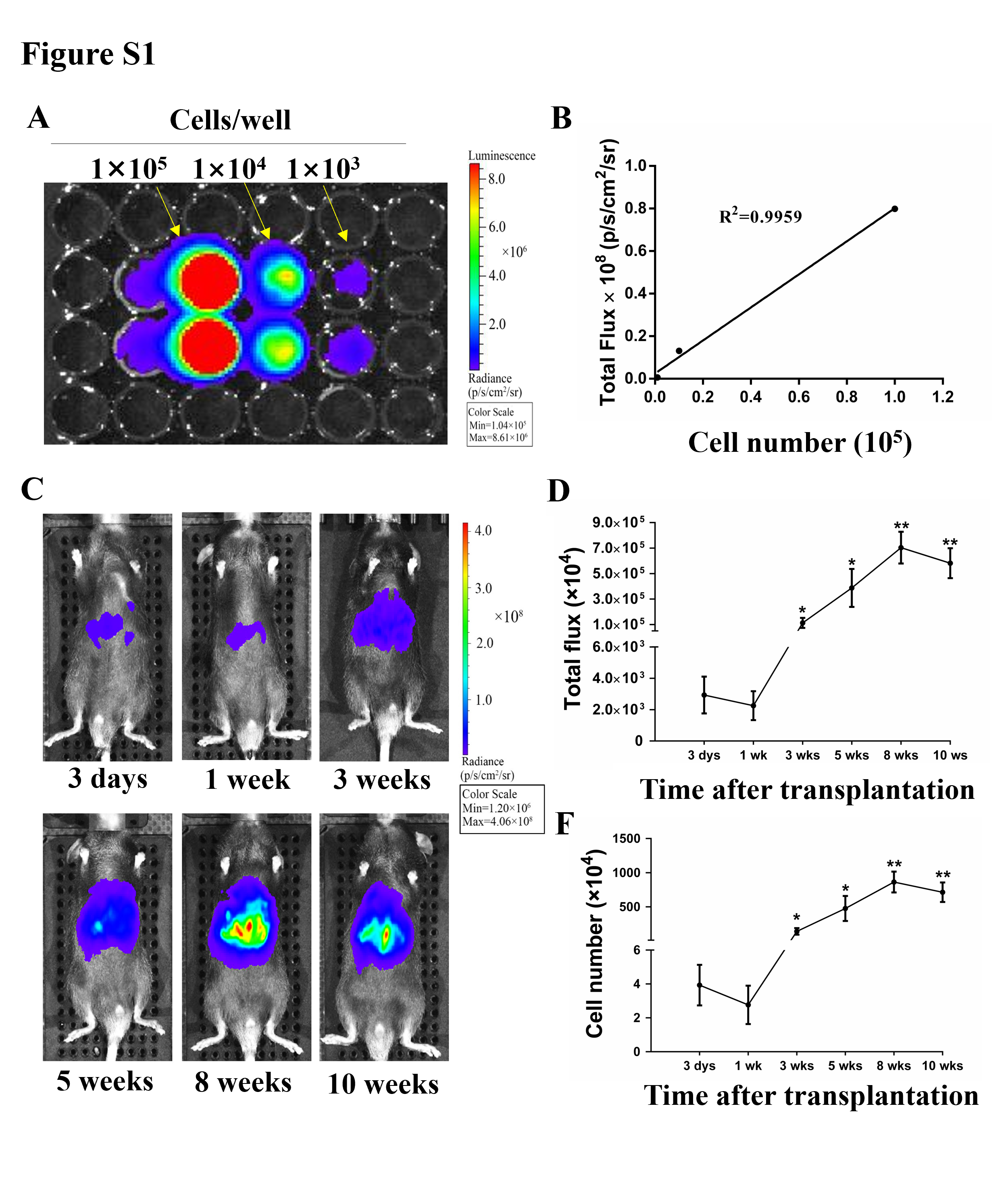

Supplement: Supplementary Figure 1 — Analysis of bioluminescence intensity of luciferase hepatocytes in vitro and in vivo after transplanting into Fah–/– mice. (A) BLI image of luciferase hepatocytes in vitro. (B) The linear correlation assay between the number of luciferase hepatocytes and BLI signal intensity, respectively. (C) Representative pictures of 2D BLI imaging of Fah–/– mice (3 days, 1, 3, 5, 8, and 10 weeks). (D) Quantified analysis of 2D BLI signal intensity of Fah–/– mice in vivo. (E) Quantified analysis of transplanted hepatocytes in 2D of Fah–/– mice in vivo. Data are shown as mean ± SEM. ∗p < 0.05, ∗∗p < 0.01, ∗∗∗p < 0.001. N = 3. Student’s t-test. [file Image_1.TIF]

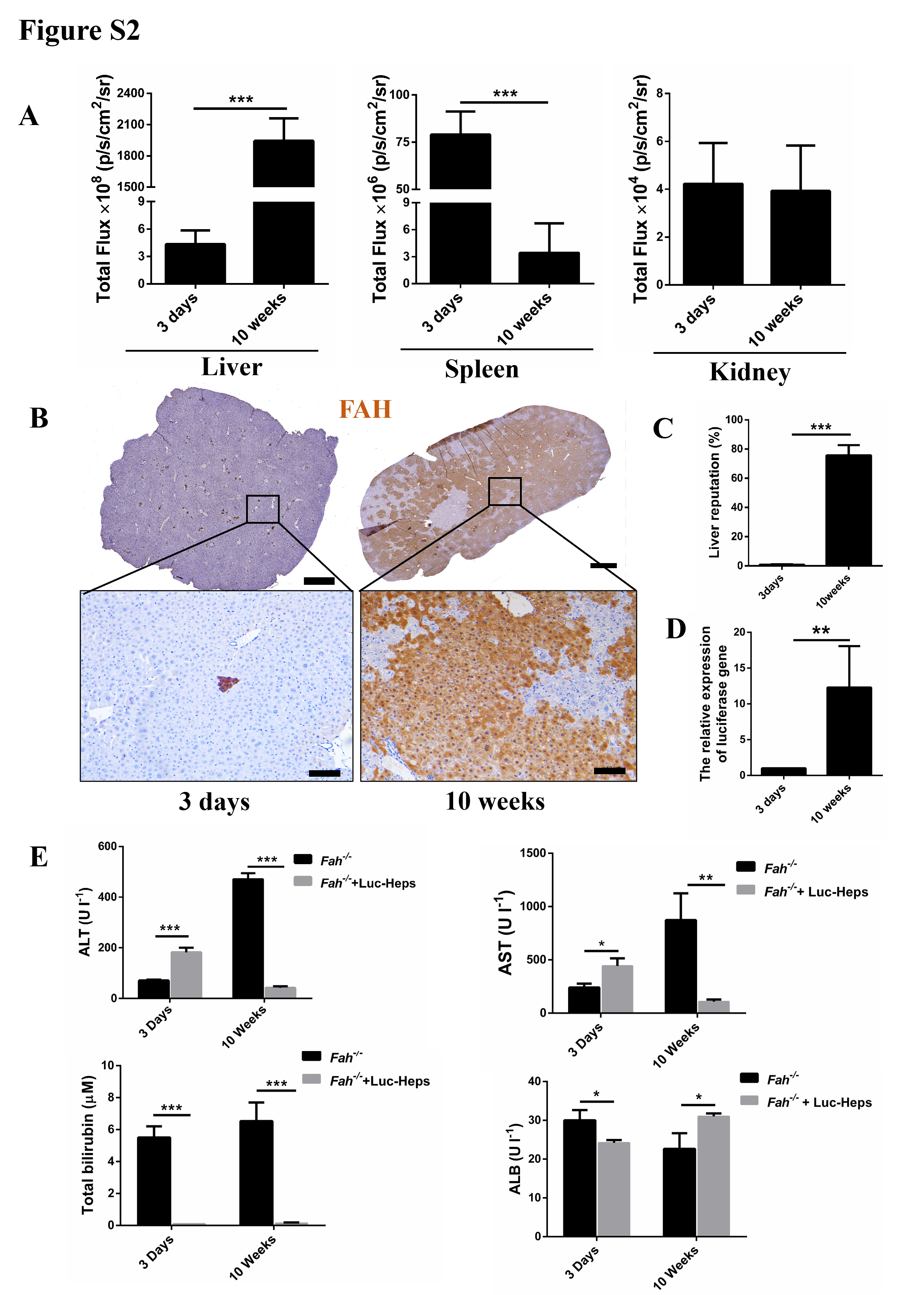

Supplement: Supplementary Figure 2 — Assessment of posttransplantation hepatocytes in Fah–/– mice. (A) Bioluminescence intensity of isolated livers, spleen, kidney of Fah–/– mice, respectively. (B) Immunohistochemistry staining of histological sections of the livers from Fah–/– mice with luciferase-hepatocyte transplantation. N = 3. Scale bars, 200 μm (up) and 50 μm (down). (C) Quantification analysis for liver regeneration of Fah–/– mice at 3 days and 10 weeks after transplantation, respectively. (D) The relative expression level of luciferase gene in the liver of Fah–/– mice. (E) Serum levels of ALT (a), AST (b), ALB (c), and total bilirubin (d) in Fah–/–, Fah–/– + luciferase hepatocytes (Fah–/– + Luc-Heps). Data are presented as mean ± SD. ∗p < 0.05, ∗∗p < 0.01, ∗∗∗p < 0.001. N = 3. Student’s t-test. [file Image_2.TIF]
